# A novel combined intelligent algorithm prediction model for the tunnel surface settlement

**DOI:** 10.1038/s41598-023-37028-w

**Published:** 2023-06-17

**Authors:** You Wang, Fang Dai, Ruxue Jia, Rui Wang, Habibullah Sharifi, Zhenyu Wang

**Affiliations:** 1 School of Civil Engineering, Central South University, Changsha, 410075 China; 2 Changsha Yaosen Engineering Technology Co., Ltd., Changsha, 410075 China

**Keywords:** Civil engineering, Mathematics and computing

## Abstract

To ensure the safety and stability of the shield tunnel construction process, the ground settlement induced by the shield construction needs to be effectively predicted. In this paper, a prediction method combining empirical mode decomposition (EMD), chaotic adaptive sparrow search algorithm (CASSA), and extreme learning machine (ELM) is proposed. First, the EMD is used to decompose the settlement sequence into trend vectors and fluctuation vectors to fully extract the effective information of the sequence; Second, the sparrow search algorithm is improved by introducing Cubic chaotic mapping to initialize the population and adaptive factor to optimize the searcher’s position formula, and the chaotic adaptive sparrow search algorithm is proposed; Finally, the CASSA-ELM prediction model is constructed by using CASSA to find the optimal values of weights and thresholds in the extreme learning machine. The fluctuation components and trend components decomposed by EMD are predicted one by one, and the prediction results are superimposed and reconstructed to obtain the predicted final settlement. Taking a shield interval in Jiangsu, China as an example, the meta-heuristic algorithm-optimized ELM model improves the prediction accuracy by 10.70% compared with the traditional ELM model. The combined EMD-CASSA-ELM prediction model can greatly improve the accuracy and speed of surface settlement prediction, and provide a new means for safety monitoring in shield tunnel construction. Intelligent prediction methods can predict surface subsidence more automatically and quickly, becoming a new development trend.

## Introduction

With the development of urban underground space, the shield construction method has become a common tunneling method. The shield tunneling process usually leads to changes in the stress state of the soil around the tunnel, and the deformation generated by the soil disturbance is transferred to the ground surface, which eventually forms surface settlement. Therefore, the prediction of surface settlement caused by shield construction is a prominent guarantee for the safety of the construction process and the stability of the surrounding environment.

Many factors and complex interactions that induce surface settlement make it difficult to predict surface settlement. At present, surface settlement prediction methods include three main categories: empirical formulas, numerical simulations, and machine learning. For single-hole tunnels, empirical formulas such as the peck formula are often improved to predict surface settlement^1–3^. But the existing empirical or semi-empirical methods can hardly take into account the external factors affecting soil deformation comprehensively, making it difficult to predict surface settlement accurately. Numerical simulation methods are commonly used as research tools in tunnel excavation problems^4^. Nevertheless, the establishment of computational models for numerical simulation requires the support of a large number of soil parameters and construction data, in high requirement for operators and computers. With the development of artificial intelligence, machine learning has been widely used in studies related to landslide displacement and subgrade settlement prediction. Yan and Ashraf^5^ used BP neural network algorithm to predict landslide displacements and found that the algorithm achieved 94.7% accuracy in landslide warning with good prediction accuracy and stability. Zhou et al.^6^ used a fusion of three intelligent algorithms, wavelet transform, artificial bee colony, and kernel extreme learning machine, to predict landslide displacements. This combined model was compared with a support vector machine model and it was found that the proposed method achieved better results in terms of accuracy and stability methods. Song et al.^7^ conducted a study on foundation pit settlement and used the PSO algorithm to optimize the settlement prediction method of SVM in order to improve the accuracy of foundation pit settlement prediction. The results showed that the model can fit the actual situation better.

The main modeling methods used in surface settlement prediction problems are a single prediction model and a combined prediction model. The main machine learning methods for single prediction models of surface settlement caused by shield tunneling include neural networks, support vector machines, random forests, and extreme learning machines. Zhang et al.^8^ comprehensively compared the performance of five machine learning algorithms, namely back-propagation neural network (BPNN), general regression neural network (GRNN), extreme learning machine (ELM), support vector machine (SVM), and random forest (RF), in predicting ground subsidence. They then used the metaheuristic algorithm particle swarm optimization (PSO) to integrate machine learning (ML) models, improving the robustness of the ML models.

Extreme learning machine (ELM), as a new neural network algorithm with strong and fast learning ability, is commonly used in short-term wind power prediction. Chen et al.^9^ used principal component analysis, tree seeding algorithm, and extreme learning machine to compose a short-term compliance prediction model for power compliance prediction and achieved good results. Xia and Wang^10^ proposed a combined IMVO-ELM prediction model to improve the prediction accuracy of short-term wind speed. Li^11^ creatively applied the ELM to predict the ground settlement induced by tunnel construction, and used a genetic algorithm (GA) to find the optimal input weights and hidden layer thresholds, and found that the optimized ELM model has a better prediction effect. Han et al.^12^ used the sine algorithm to optimize the regularized extreme learning machine and used the model to predict ground subsidence around excavation pits. They found that the model had high accuracy and generalization ability. Zhang et al.^13^ used a machine learning model to predict the ground settlement caused by shield tunnels. By considering geological conditions, construction parameters, and construction sequence, a hybrid neural network model was developed to analyze and predict the ground settlement in shield tunnels, and the results verified the accuracy of the proposed hybrid model.

Combined prediction models for ground settlement caused by shield tunneling include the combination of metaheuristic algorithms and single prediction models, and the combination of decomposition algorithms and single prediction models. Liu et al.^14^ compared four different metaheuristic algorithms, differential evolution (DE), particle swarm optimization (PSO), genetic algorithm (GA) and ant colony optimizer (ACO), for optimizing adaptive neuro-fuzzy inference systems (ANFIS). It was found that the use of particle swarm optimization was more effective and the PSO-ANFIS model was used to predict the ground settlement of the shield tunnel with good results. Qiao et al.^15^ proposed a combination prediction model called a grey wolf optimization algorithm and extreme learning machine (GWO-ELM) to predict ground subsidence caused by excavation pits in different influencing states. They found that the predicted results were good. Zhang et al.^16^ proposed a combination prediction model called DQN-PSO-ELM, which achieved good results in predicting the ground response caused by tunneling in real time. Li et al.^17^ proposed a combined CPSO-SVM prediction model using particle swarm algorithm and chaotic mapping optimized support vector machine. The model is used to predict the deformation of the tunnel envelope body, which can better describe the relationship between deformation and time. Using metaheuristic optimization algorithms to improve single-prediction models has become an effective way to improve single-model prediction accuracy. However, the choice of which optimization algorithm to use for the specific problem of surface settlement caused by shield tunneling still needs to be resolved.

Nature-inspired meta-heuristic algorithms have gained a great deal of attention in the last decade. Mehood et al.^18^ proposed an improved chaotic gray Wolf optimizer method based on the Gray Wolf algorithm, which incorporates logical chaotic mapping and improved convergence factors in GWO. In 2022, a meta-heuristic algorithm based on the Dwarf Mongoose Optimization Algorithm (DMOA) was proposed for parameter estimation of autoregressive exogenous (ARX) models^19^. It was found that DMOA has advantages in convergence speed, estimation accuracy, robustness and reliability in ARX identification. In addition, Mehood et al.^20,21^ applied the Aquila optimization algorithm and the Marine predator optimization algorithm using keyword separation technology in different fields in 2022. The development of meta-heuristic algorithm also promotes the development of surface subsidence prediction. Sparrow search algorithm (SSA) is a new type of swarm intelligence metaheuristic optimization algorithm that can effectively optimize parameters in other single prediction algorithms. It is more suitable for solving continuous variable optimization problems like the surface settlement. Li and Qiu^22^ proposed an SSA-ELM model for predicting open-pit slope displacement and found that the prediction accuracy was higher than that of the BPNN model. Li et al.^23^ analyzed and compared the optimizing effects of three optimization algorithms, SSA, GA, and PSO, combined with BPNN prediction, and found that SSA had a higher optimization efficiency and could more accurately predict ground subsidence caused by excavation pits. Whereas, due to the drawbacks in population initialization, position update strategy, etc., SSA has the problems of weak global search ability, slow convergence speed, and easy fall into local optimality. Some scholars also improve the SSA in various ways. Zhu et al.^24^ used a random wandering strategy to randomly perturb the sparrow to improve its search ability, and added Gaussian mutations to the iterative process of the sparrow to enhance the local search ability. Yang et al.^25^ introduced the chaotic mapping strategy and the adaptive weighting strategy to propose a new adaptive SSA, which can obtain higher convergence accuracy, faster convergence speed, and better diversity and exploration ability.

In addition to the combination of metaheuristic algorithms and single prediction models, the combination of decomposition algorithms and single prediction models is also a common type of combined prediction model. Kaloop et al.^26^ proposed a Wavelet-PSO-ELM combined prediction model. They used wavelet algorithms to decompose and optimize the Extreme Learning Machine prediction model for predicting wave height and achieved good results.

Empirical mode decomposition (EMD) is an adaptive decomposition algorithm that can effectively achieve the transformation from nonlinear to linear and nonsmoothed to smooth processing. Cao et al.^27^ used fully integrated EMD to divide the one-dimensional ground subsidence data into multidimensional ones, and each component was predicted by the LSTM neural network and superimposed to obtain the final prediction results, which produced better prediction results. Luo et al.^28^ proposed a combined prediction model based on empirical mode decomposition (EMD), support vector regression (SVR) and wavelet neural network (WNN) called the EMD-SVR-WNN model. The EMD method was used to decompose structural monitoring data, and then the WNN and SVR methods were used to make predictions. Experimental results showed that this combined model was able to accurately predict structural settlement. At present, the combined model has been widely used.

The emergence of intelligent algorithms has provided new ideas and means for subsidence prediction, which is a good complement to traditional subsidence prediction methods. However, different algorithms have their limitations, making it difficult to predict surface settlement values accurately and independently. The combined prediction method enhances the robustness and adaptability of the research object and analyzes the surface settlement change pattern from multiple perspectives, and then establishes a systematic prediction model with powerful integrated processing information and higher prediction accuracy. Intelligent prediction methods can predict surface subsidence more automatically and quickly, becoming a new development trend.

This paper proposes a combined prediction model of empirical mode decomposition (EMD), chaotic adaptive sparrow search algorithm (CASSA), and extreme learning machine, which is more targeted to solve the continuous variable optimization prediction problem of surface settlement. It is decomposed into trend and fluctuation components to fully extract the effective information of the sequence. The CASSA is proposed by introducing Cubic chaotic mapping to initialize the population and adaptive factor to optimize the searcher's position formula to improve the sparrow search algorithm. The ELM is modified with optimized parameters to predict the decomposed fluctuation components and trend components one by one. The two predictive components are superimposed and reconstructed to complete the surface settlement prediction of the shield tunnel. And the prediction results are compared with the traditional model to evaluate the prediction accuracy of the model.

## Methodology

### Empirical mode decomposition

**Figure 1:**
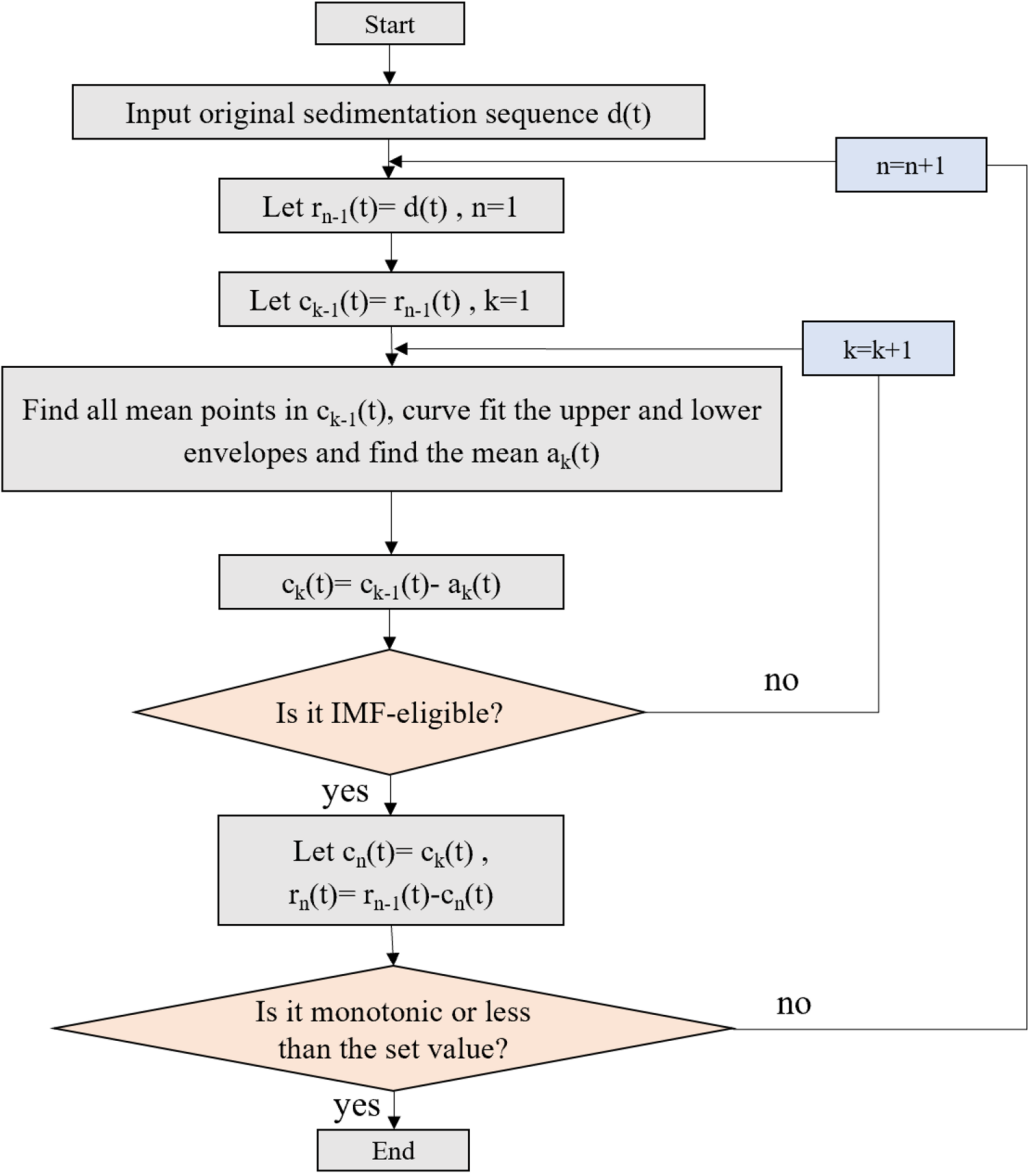
EMD flow chart.

Empirical mode decomposition is a method that can adaptively realize the transformation from nonlinear to linear, and can decompose surface settlement into trend and fluctuation components to fully extract subsidence information. The empirical mode decomposition can better decompose such nonlinear problems as surface settlement in shield tunnels. The surface settlement original series is decomposed into several intrinsic mode functions (IMFs) by empirical mode decomposition, and each IMF component represents the variation pattern of the original series at each time scale, and the original series is effectively decomposed, which highlights the series characteristics^29^. Surface settlement time series are usually non-smooth nonlinear time series, which can be disintegrated into trend and fluctuation terms by EMD. The fluctuation term reflects the volatility pattern of the surface settlement original series, while the trend term reflects the stable direction of the series and presents the general trend of the series change. The essence of the original EMD sequence of surface settlement is to decompose a non-stationary nonlinear sequence into a finite number of IMF components and a trend term. The process of decomposition to obtain the IMF is shown in Fig. 1. In which the original subsidence sequence is represented by d(t), the residual sequence is represented by r(t), and each obtained intrinsic mode function (IMF) component is represented by c_i_(t).

The empirical mode decomposition steps are specified as follows.

1. The original subsidence sequence monitored in the field is *d*(*t*), to obtain all the maximum and minimum points of *d*(*t*), we used the third sample interpolation function to do the upper envelope curve for all maximum and minimum points to do the envelope curve, and then solve for the average value of the upper and lower envelopes *a*_1_(*t*).
2. The original settling sequence* d*(*t*) is subtracted from the average of the upper and lower envelope curves *a*_*1*_*(t)* to obtain the new sequence *c*_1_(*t*).
  1 $${c}_{1}\left(t\right)=d\left(t\right)-{a}_{1}(t)$$
3. To assess whether the new sequence *c*_1_(*t*) meets the IMF condition or not, if it does, *c*_1_(*t*) is the first IMF component. If not, *c*_1_(*t*) is considered as the original sequence repeat the above steps until the IMF conditions are met and the first IMF component is obtained, and the residual sequence is *r*_1_(*t*)*.*
  2 $${r}_{1}\left(t\right)=d\left(t\right)-{c}_{1}(t)$$
  The IMF condition is that the difference in values between the number of extreme points and the number of over-zero points cannot be greater than 1. The average value between the upper envelope corresponding to the local maximum and the lower envelope corresponding to the local minimum at the position of the number axis at any point in time for the sink value should be zero.
4. The residual sequence *r*_1_(*t*) is continued to be decomposed according to step (1) (2) until the nth IMF component is obtained, and the residual sequence *r*_*n*_(*t*) is monotonic or less than the set value, and the decomposition ends here. The residual sequence *r*_*n*_(*t*) can reflect the general trend of the original settling sequence, while the individual IMF components *c*_*i*_(*t*) decomposed can reflect the fluctuation of the original settling sequence. The original sedimentation sequence can be written as Eq. (3) after empirical mode decomposition.
  3 $$d\left(t\right)=\sum_{i=1}^{n}{c}_{i}(t)-{r}_{n}(t)$$

### Chaotic adaptive sparrow search algorithm

With the rapid development of metaheuristic algorithms, the Skyhawk optimization algorithm, Ocean predator algorithm, and Sparrow search algorithms, show excellent results in the field of intelligent algorithms such as optimization extreme learning machine. Using different optimization algorithms for different problems will achieve better results. The Skyhawk optimization algorithm is suitable for solving multi-objective optimization problems, while the Marine Predator algorithm is an optimization technique developed based on the behavioral characteristics of marine biological predators and is suitable for solving discrete optimization problems. The sparrow search algorithm is more suitable for solving continuous variable optimization prediction problems such as surface sedimentation.

The sparrow search algorithm is a new intelligent group algorithm proposed by Xue and Shen^30^ in 2020, which simulates the behavior of a sparrow flock foraging and escaping predators and proposes a group intelligent optimization algorithm with a simple structure and obvious advantages in terms of convergence speed and optimization-seeking accuracy. By observing the predatory and anti-predatory behaviors of sparrows, it is proposed that sparrows will continuously seek the optimal position to ensure safety during predation and anti-predation. Using the sparrow search algorithm, the position updates of discoverers and joiners can be used to optimize for the parameters in the extreme learning machine. However, the sparrow algorithm has some flaws in population initialization and position upgrade strategy. This will lead to poor global search ability, slow convergence, and easy to fall into local optimum. Therefore, this paper improves the sparrow search algorithm by using Cubic chaotic mapping and introducing adaptive factors to build a chaotic adaptive sparrow search algorithm in turn. It improves its global search capability and convergence speed.

The traditional sparrow search algorithm computing process consists of 3 parts: discoverers, joiners, and vigilantes, where the total number and proportion of discoverers and joiners are constant. The two can be transformed into each other according to the change in fitness value. The optimal position of the population members is continuously updated by predation and anti-predation behaviors. Let’s consider the population size to be n. In the t iteration, the position of the discoverer is updated in the way of Eq. (4).

4 $$ x_{i,j}^{t + 1} = \left\{ {\begin{array}{*{20}c} {x_{i,j}^{t} \cdot exp\left( { - \frac{i}{{\alpha \cdot iter_{max} }}} \right),} & {R_{2} < ST} \\ {x_{i,j}^{t} + Q \cdot L,} & {R_{2} \ge ST} \\ \end{array} } \right. $$

where $\alpha$ represents the random number between (0,1]; ${iter}_{max}$ represents the total number of iterations; Q represents a random number obeying normal distribution; L is a matrix of 1 × D and all values are 1, where D is the dimension of the space; ${R}_{2}$ is the alarm value, which is a random number between [0,1]; ST is the safety threshold. ST ∈ [0.5,1].

When ${R}_{2}<ST$, the individuals of the sparrow search algorithm converge to the optimal solution by moving closer to the zero point. The position of the individual is getting smaller after each iteration, and the local search ability is stronger near the zero point. This also leads to the lack of search range and global search ability in the early stage of the sparrow algorithm, so the optimal solution at non-zero is easily missed.

To solve the problem of insufficient search range and problem in the early stage, Cubic chaotic mapping is introduced to initialize the population while the adaptive factor is introduced to optimize the searcher's position formula for improvement to solve the problem of poor global search capability. Specifically, the new developments made to the sparrow search algorithm in this paper include the following two main parts.

1. Cubic chaotic mapping initializes the population

In the traditional sparrow search algorithm, the population members are generated randomly within the specified search range when the population and fitness are initialized. This may lead to low uniformity of distribution of the initial population within the search range. However, chaotic motion is random, regular, and ergodic, so chaotic mapping can be used to initialize the population members. It makes the population more uniformly distributed in the search range and improves the quality of the initial population distribution.

**Figure 2:**
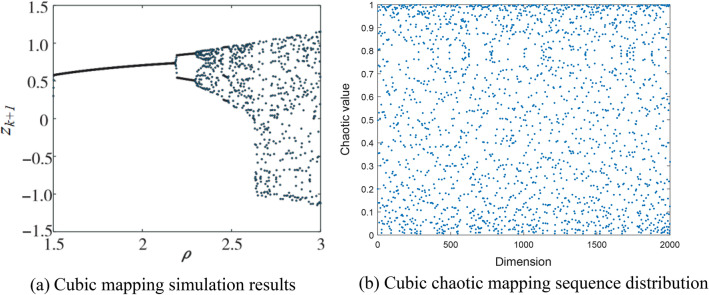
Cubic chaotic mapping test results.

Cubic chaotic mapping is a representation and a widely used class of models^31–33^. Its mathematical expression is Eq. (5).

5 $$ z_{k + 1} = \rho z_{k} \left( {1 - z_{k}^{2} } \right) $$

where $\rho$ is the chaotic mapping coefficient. For different $\rho$ values, choose the best value for $\rho$ according to the Lyapunov Exponent graph^34^. Among them, $\rho$ is the control parameter, and the value of parameter $\rho$ is (1.5,3). The simulation was carried out according to Eq. (5), and the simulation results were shown in Fig. 2a. When the initial value of z_0_ is (0,1), and z_0_ = 0.315, $\rho$ = 2.595, Cubic mapping has good chaotic ergodic property. Figure 2b shows the Cubic chaotic mapping sequence distribution under 2000 iterations when $\rho$ = 2.595. The test results of Cubic chaotic mapping are shown in Fig. 2b, which shows that the improvement of the population with Cubic chaotic mapping can have a higher search accuracy and better stability.

(2) Adaptive factor optimization of discoverer position

As the search range changes, the search ability of the population members should change relatively. At the beginning of the iteration, the population members move in a large range and need a higher search capacity to check in a large range. And with continuous iteration, the search range of the local optimal solution decreases, then the local search ability of the algorithm should be further optimized.

Therefore, an adaptive factor is added to the discoverer position update formula to optimize the algorithm performance. The size of the adaptive factor weights changes with the number of iterations. At the beginning of the algorithm operation, the weights are larger, and the algorithm has a strong global search capability and converges quickly. When the optimal solution is approached, smaller weights can search exactly in a small area.

The improved discoverer formula is shown in Eq. (6).

6 $$ x_{i,j}^{t + 1} = \left\{ {\begin{array}{*{20}c} {x_{i,j}^{t} + \lambda \cdot \alpha \left( {x_{i,j}^{t} - f_{g} } \right),} & {R_{2} < ST} \\ {x_{i,j}^{t} + Q \cdot L,} & {R_{2} \ge ST} \\ \end{array} } \right. $$

where $\lambda = \sin ((\pi \cdot t))/(2.T)$, and ${f}_{g}$ is the best fitness of the current population.

Equation (6) used sine function in mathematics for iterative optimization, and improves the traditional sparrow search algorithm. According to the discoverer position update formula of sparrow search algorithm, when ${R}_{2}<ST$, each dimension of the discoverer is getting smaller; when ${R}_{2}\ge ST$, the discoverer will randomly move to the current position according to the normal distribution. As a result, the algorithm tends to approach the global optimal solution at the beginning of iteration, which easily leads to premature convergence of the algorithm and local optimal. Therefore, this paper introduced adaptive factor $\lambda$ to improve the discoverer position update formula in sparrow search algorithm.

### Extreme learning machine

**Figure 3:**
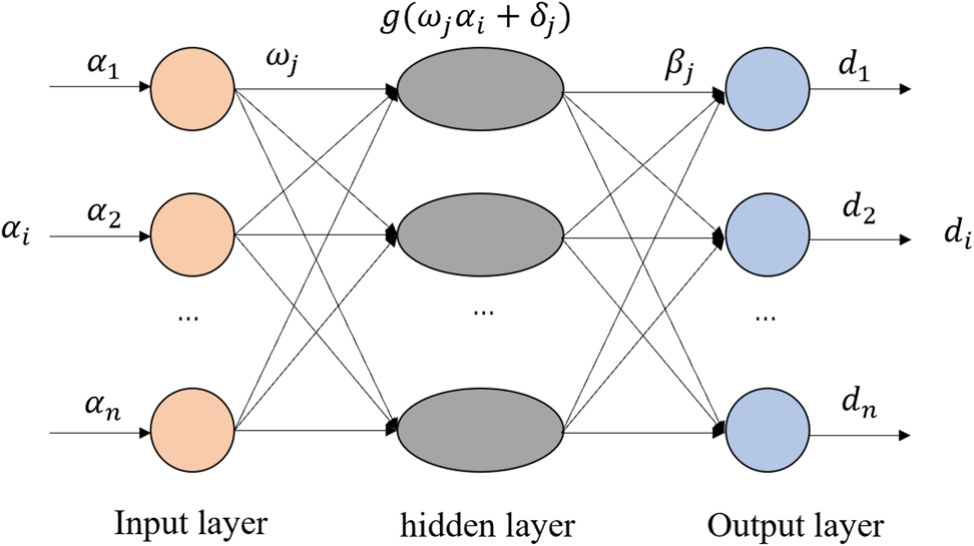
Extreme learning machine training structure.

The Extreme Learning Machine was proposed by Professor Huang in 2004, and ELM is a single implicit layer feedforward neural network algorithm^35–37^. The training structure of the Extreme Learning Machine is shown in Fig. 3. Compared with the commonly used BP neural network, ELM only requires a random selection of initial weights and thresholds, unlike BP which is a back propagation algorithm to adjust the weights and thresholds among the layers, thus reducing the learning time and structural complexity of the algorithmic model and improving the overall training speed of the model. Prediction of surface subsidence using ELM as part of a combined model can improve prediction efficiency.

The connection weights between the input layer and the hidden layer and the threshold value of the hidden layer of the extreme learning machine are randomly generated during training and learning, and are not adjusted during subsequent network training. The unique optimal solution is obtained mainly by setting the number of neurons in the hidden layer. A typical single implicit layer feedforward neural network can be represented as:

7 $$\sum_{j=1}^{l} {\beta }_{j}g({\omega }_{j}{\alpha }_{i}+{\delta }_{j})= {d}_{i}$$

Of which,

8 $$ \omega_{j} = \left[ {\omega_{j1} ,\omega_{j2} , \ldots ,\omega_{jn} } \right]^{T} $$

9 $$ \beta_{j} = \left[ {\beta_{j1} ,\beta_{j2} , \ldots ,\beta_{jn} } \right]^{T} $$

where ${\omega }_{j}$ is the connection weight from the input layer to the j node of the hidden layer; ${\beta }_{j}$ is the output weight connecting the j hidden layer node to the output node; ${\delta }_{j}$ is the threshold of the j hidden layer node; ${\alpha }_{i}$ is the input of the network; ${\omega }_{j}{\alpha }_{i}$ is the inner product of ${\omega }_{j}$ and ${\alpha }_{i}$; $g(x)$ is the activation function; ${d}_{i}$ is the output of the network.

### Construction of a combined EMD-CASSA-ELM intelligent model

**Figure 4:**
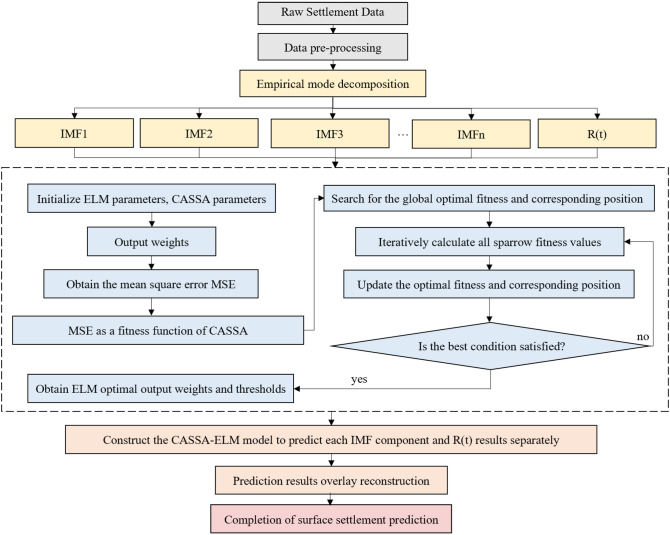
Combined EMD-CASSA-ELM prediction mode.

In this paper, we propose a combined EMD-CASSA-ELM intelligent prediction model. In this new combined model, the extreme learning machine is optimized using the sparrow search algorithm and empirical mode decomposition. The EMD-CASSA-ELM model is proposed by combining the metaheuristic algorithm, the adaptive decomposition algorithm, and the single prediction model. Besides, two parts of the traditional sparrow search algorithm are improved. First, the initialized population is improved using cubic chaotic mapping to improve the quality of the initial population. Secondly, the location update strategy is improved using adaptive factors to improve the global search capability and convergence speed of the algorithm. The model construction process is shown in Fig. 4.

The EMD-CASSA-ELM combined prediction model is constructed as follows.

1. Input initial settlement data and perform data pre-processing. The initial settlement data are normalized. The field monitoring data usually contains some special samples due to monitoring errors, and the special samples usually contain abrupt variability characteristics. The special samples tend to cause overfitting in the model, so the special samples need to be removed and supplemented by linear interpolation. In addition, the normalization process is needed to improve the training speed and training accuracy, and the normalized sample settlement value *d’* is shown in Eq. (10).
  10 $$ d_{i}^{\prime} = \alpha + \beta \frac{{d_{i} - d_{min} }}{{d_{max} - d_{min} }}\quad i = 1,2, \ldots ,n $$
  where $\alpha$ and $\beta$ are normalization coefficients; ${d}_{max}$ and ${d}_{min}$ are the maximum and minimum settlement values in the sample; ${d}_{i}$ is the surface settlement value at $i$ time.
2. The initialized surface sedimentation series are decomposed by EMD. The EMD decomposition method is used to decompose the surface sedimentation series into a trend component R(t) representing the development of surface sedimentation and some smooth fluctuation IMF components, to better obtain the intrinsic information about the surface time series.
3. Initialize ELM parameters and CASSA parameters. Set the number of ELM hidden layers, activation function, CASSA population number, the maximum number of iterations, and calculation dimension.
4. Model building and training. Using the input samples and the determined initialized ELM parameters and CASSA parameters, the model is trained. The root mean square error MSE of the training output is used as the fitness function of CASSA. The smaller the MSE error, the better the fit of the predicted data to the original data. The fitness function is expressed as Eq. (11).
  11 $$fitness={min(MSE}_{predict})$$
5. Search the global optimal fitness and the corresponding position of CASSA, and iteratively calculate the fitness values of all sparrows. If the optimal conditions are met, the weights and thresholds of the optimized ELM are output. If the optimal conditions are not met, continue to iteratively calculate the fitness values of all sparrows and update the optimal fitness and optimal positions until the optimal conditions are met. The output optimal weights and thresholds are brought into the ELM prediction model to construct the CASSA-ELM prediction model.
6. The components of the empirical mode decomposition are predicted one by one using the CASSA-ELM prediction model, and the predictions are overlaid and reconstructed. The predicted final surface subsidence is obtained.

## Case analysis

### Project overview

**Figure 5:**
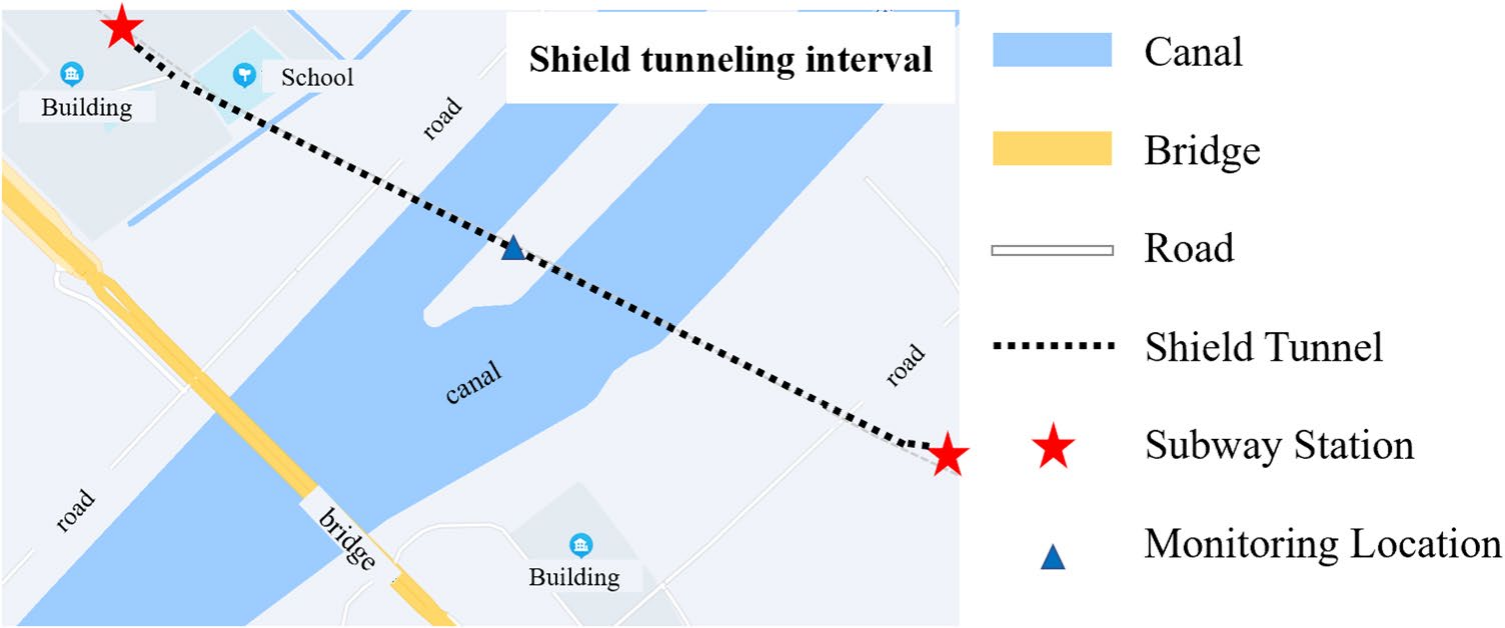
Shield construction plan.

Take a subway shield tunnel in the eastern coastal region of China as an example. The shield tunnel goes under the river. The minimum net distance between the tunnel and the bottom of the canal on the west side of the island is 9.9 m, while the minimum net distance between the tunnel and the bottom of the canal on the east side of the island is 12.69 m. The span of the river reaches 414 m. The construction plan of the shield is shown in Fig. 5.

**Figure 6:**
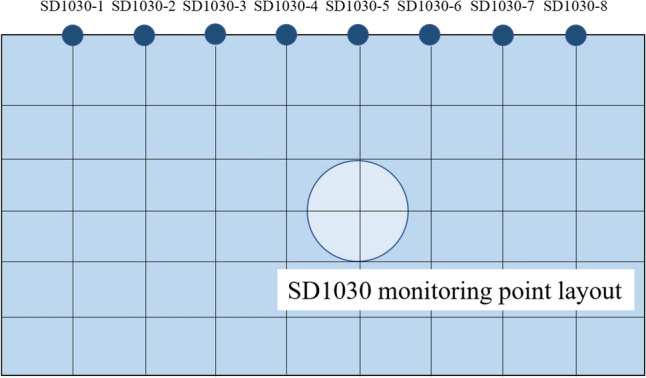
SD1030 section monitoring point layout.

The left line of the interval is 1789 m long, with a total of 1491 rings, and the right line is 1806 m long, with a total of 1505 rings. The left and right lines cross the river at rings 820–1165 and 836–1179 respectively. Measurement points were arranged around the river to monitor its surface settlement, where the significant settlement occurred surrounding. The shield completely passed through the river on May 25th, 2020, and the monitoring time spanned from May 2nd, 2020, to October 9th, 2020, with one monitoring data obtained every day. The arrangement of measurement points is shown in Fig. 6.

**Figure 7:**
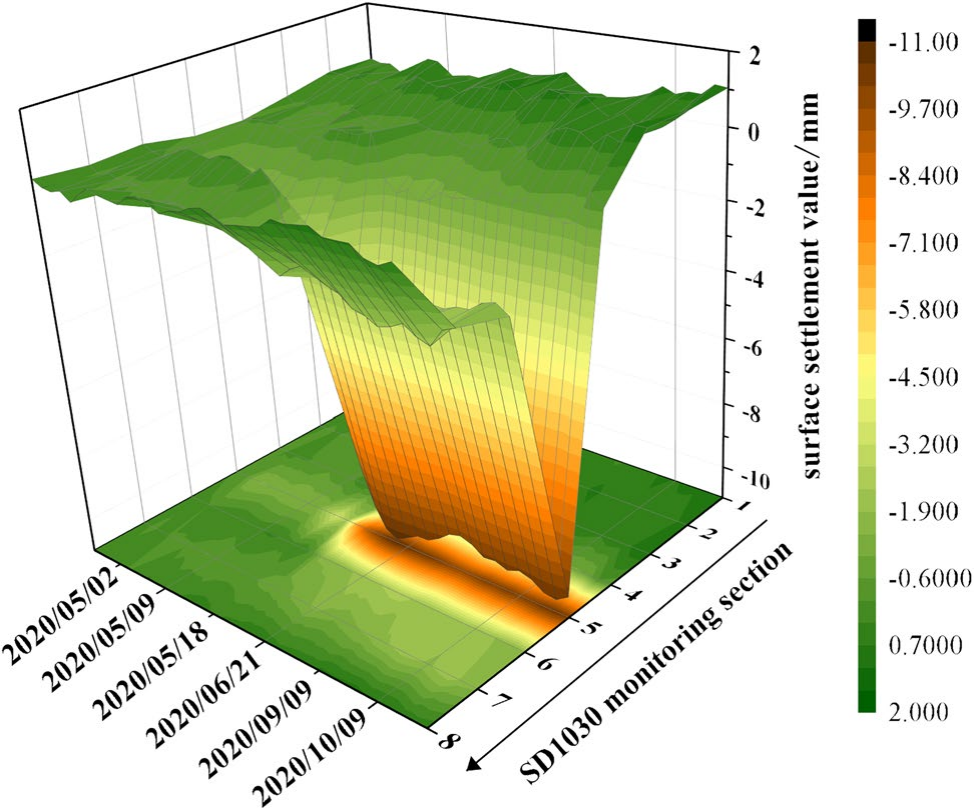
Site monitoring of settlement changes at section SD1030.

As shown in Fig. 7, it can be seen that the settlement amount and settlement speed of the monitoring point above the axis reach the maximum on the same monitoring section during the shield tunnel underpass, while the settlement amount gradually decreases to both sides of the axis. The settlement amount at the monitoring point SD1030-5 directly above the shield machine is selected as the model prediction sample. The change of surface settlement at SD1030 in the right line section is shown in Fig. 7.

**Figure 8:**
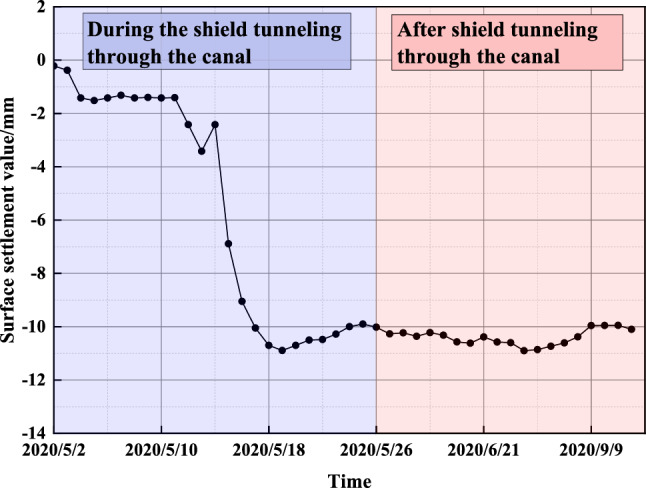
Site monitoring of settlement changes at SD1030-5.

The settlement changes at SD1030-5 are shown in Fig. 8, which can be divided into the settlement phase of the shield through the canal and the stabilization phase of the shield after passing the canal.

### Data processing and prediction

**Figure 9:**
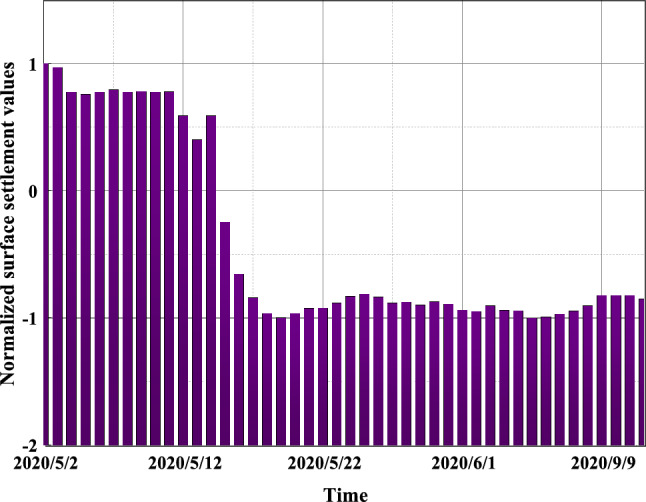
The normalized results of the sedimentation sequence.

To eliminate the differences in data magnitudes, the data needs to be normalized beforehand. This can make surface settlement prediction morereliable and scientific. In this paper, the Max–Min normalization method, which is suitable for ELM, is chosen. The main process is to perform a linear transformation on the original sequence and adjust the sequence data to the range of [− 1,1]. The raw settlement time series data monitoring point SD1030-5 was normalized, and the normalized results obtained are shown in Fig. 9.

**Figure 10:**
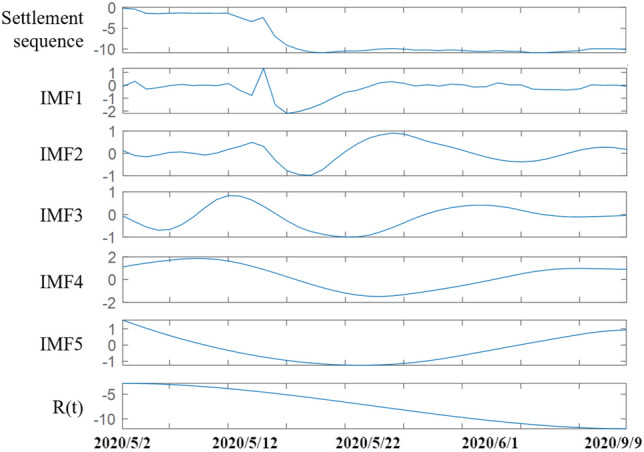
Empirical mode decomposition results.

The surface subsidence time series data in the monitoring samples are non-stationary signals and are affected by multiple factors with certain randomness and abrupt variability. EMD is used to decompose the original sedimentation series data to obtain IMF components and residual components, which are used to highlight the characteristics of the original series. The EMD decomposition of the surface sedimentation sequence of measurement point SD1030-5 was performed to obtain five IMF components and one residual component. The five IMF components are used as the fluctuation components of the surface settlement variance, and the residual component is used as the trend component, as shown in Fig. 10.

**Figure 11:**
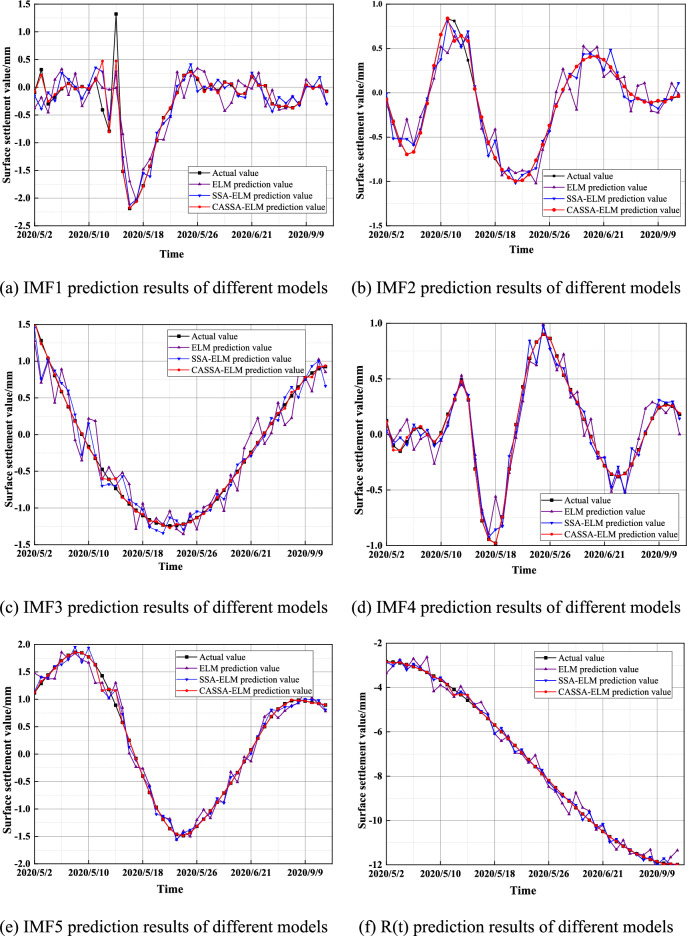
Comparison of prediction results of each component.

The five IMF components and one residual component obtained from the decomposition are respectively subjected to the CASSA-ELM prediction. The first 80% of the data of each IMF component was selected as the input value of the corresponding component, and the CASSA-ELM model was used for training. And the last 20% of the data was used for validation of the trained model. The number of ELM implicit layers was set to 20, and the activation function was the Sigmoid function. The maximum number of iterations of CASSA was 50, the warning value ST = 0.6, the proportion of discoverers PD = 0.8, and the proportion of sparrows aware of the danger were SD = 0.2. The model was trained and learned several times by MATLAB, and the predicted results of each component were obtained after several tests, as shown in Fig. 11.

Figure 11a–e show the predicted results of the five IMF fluctuation components, respectively, and Fig. 11f shows the predicted results of the residual trend components. As seen in Fig. 11, the CASSA-ELM model results curve is similar to the actual value curve and fits and predicts better compared to the ELM model and SSA-ELM model.

**Figure 12:**
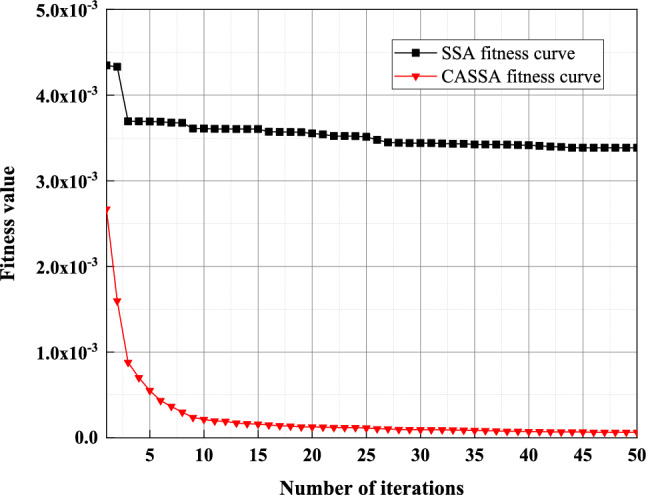
Comparison of the fitness curves of SSA and CASSA.

Figure 12 shows the adaptation curve plots of SSA and CASSA after 50 iterations. It can be seen that the best fitness curves of both SSA and CASSA have basically reached convergence at 50 iterations. Although the convergence trends of the two algorithms are close, the fitness curve of CASSA converges faster than that of SSA evident, and the stability of CASSA is better than that of SSA. The optimal solutions found by CASSA are more accurate than those found by SSA.

### Results and evaluation

**Figure 13:**
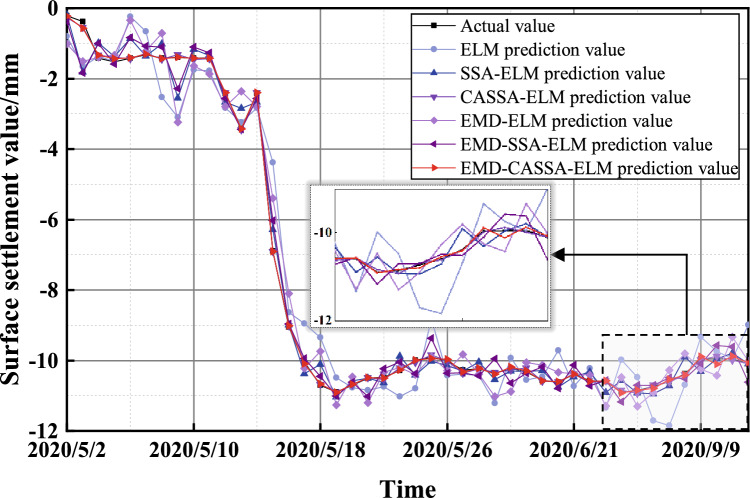
Surface settlement prediction results and model comparison.

The final prediction results are obtained by the EMD of the original data and superimposed reconstructions of each component of the decomposition after prediction one by one, as shown in Fig. 13.

Figure 13 shows the comparison of the six prediction models with the actual values. Among them, the closest prediction result to the actual value is the EMD-CASSA-ELM model, and the least close is the ELM model. The traditional ELM model has large fluctuations in surface settlement prediction results and test results, while the ELM model improved by EMD and CASSA has better fitting accuracy and prediction accuracy, which can effectively respond to the engineering reality.

**Figure 14:**
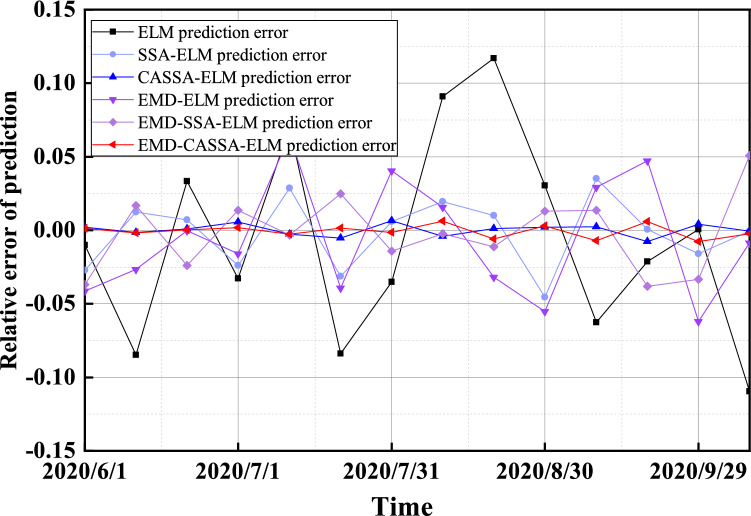
Relative error of surface settlement prediction.

Figure 14 shows the relative errors of each prediction model, and the relative error results show that the EMD-CASSA-ELM model proposed in this paper has a very stable relative error and the smallest error. Since surface settlement is caused by formation loss, there are various factors cause formation loss. The surface settlement of a shield tunnel is influenced by multiple factors such as tunneling speed, stratigraphic parameters, cutter speed, soil pressure, etc., resulting in not only regular trend settlement of the ground surface during tunneling, but also fluctuating changes based on it. Therefore, the ELM prediction model improved by EMD and CASSA can deeply explore the data information of surface settlement and make the prediction law more consistent with the surface settlement change trend.

The combined model can be evaluated by three indexes: mean square error (MSE), root mean square error (RMSE), and mean absolute percentage error (MAPE). The smaller the values of MSE, RMSE, and MAPE, the better the prediction results. The calculation formula of each accuracy evaluation index is as follows.

(1) MSE

12 $$ MSE = \frac{1}{N}\sum\limits_{i}^{N} {\left( {\mathop {d_{i} }\limits^{ \wedge } - d_{i} } \right)^{2} } $$

(2) RMSE

13 $$ RMSE = \sqrt {\frac{1}{N}\sum\limits_{i = 1}^{N} {\left( {\mathop {d_{i} }\limits^{ \wedge } - d_{i} } \right)^{2} } } $$

(3) MAPE

14 $$ MAPE = \frac{1}{N}\sum\limits_{i = 1}^{N} {\left| {\frac{{\mathop {d_{i} }\limits^{ \wedge } - d_{i} }}{{d_{i} }}} \right|} $$

where *d*_*i*_ represents the original sequence of surface settlement prediction values, $\widehat{{d}_{i}}$ represents the predicted value of surface settlement at the *i*th moment, and *N* represents the number of predicted sample points. Among them, the RMSE can assess the fluctuation of the error, and the MAPE can assess the overall accuracy.

The predicted effects of each component obtained by EMD of the settlement sequence at monitoring point SD1030-5 were compared and analyzed. And the predicted results of ELM, SSA-ELM, and CASSA-ELM models were compared to evaluate indexes, and the results are shown in Table 1.

**Table 1:** Comparative analysis of evaluation indicators.

Component	Model	MSE	RMSE	MAPE
IMF1	ELM	0.1282	0.3580	4.7890
	SSA-ELM	0.0666	0.2581	1.4449
	CASSA-ELM	0.0345	0.1858	0.2420
IMF2	ELM	0.0174	0.1321	2.8775
	SSA-ELM	0.0069	0.0829	0.7263
	CASSA-ELM	0.0001	0.0104	0.1378
IMF3	ELM	0.0302	0.1737	1.0910
	SSA-ELM	0.0118	0.1086	0.4638
	CASSA-ELM	0.0023	0.0476	0.0514
IMF4	ELM	0.0267	0.1635	0.6439
	SSA-ELM	0.0107	0.1034	0.2542
	CASSA-ELM	0.0033	0.0578	0.0168
IMF5	ELM	0.0552	0.2350	0.9755
	SSA-ELM	0.0212	0.1457	0.2692
	CASSA-ELM	0.0012	0.0342	0.0507
R(t)	ELM	0.1247	0.3531	0.0524
	SSA-ELM	0.0265	0.1629	0.0218
	CASSA-ELM	0.0028	0.0534	0.0043

As can be seen from Table 1, for each component, the MSE, RMSE, and MAPE of the CASSA-ELM prediction model are reduced compared to the ELM model and the SSA-ELM model. Among them, the MSE, RMSE, and MAPE of the CASSA-ELM prediction results from the trend component were reduced by 12.19%, 29.98%, and 4.82%, respectively, compared to the ELM model. Compared with the SSA-ELM model, they are reduced by 2.73%, 10.95%, and 1.76%, respectively. It indicates that the improved SSA-optimized ELM model is more effective for short-term surface subsidence.

The prediction results after EMD reconstruction were compared with the results without decomposition reconstruction. The MSE, RMSE, and MAPE of the six prediction models are also calculated, as shown in Table 2.

**Table 2:** Comparison of evaluation indicators of each model.

Model	MSE	RMSE	MAPE
ELM	0.6516	0.8072	0.2589
SSA-ELM	0.1525	0.3957	0.1534
CASSA-ELM	0.0030	0.0755	0.0274
EMD-ELM	0.3901	0.6246	0.2031
EMD-SSA-ELM	0.1557	0.3946	0.1050
EMD-CASSA-ELM	0.0026	0.0513	0.0144

As shown in Table 2, the MSE, RMSE, and MAPE of the decomposed-reconstructed CASSA-ELM prediction model are reduced by 0.03%, 2.42%, and 1.30%, respectively, compared with the non-decomposed-reconstructed CASSA-ELM prediction model. Compared with the traditional ELM prediction model, they are reduced by 64.90%, 75.59%, and 24.45%, respectively. The final settlement predicted by the conventional ELM model is 8.99 mm, while that predicted by the combined EMD-CASSA-ELM prediction model is 10.07 mm, which is only 0.03 mm different from the actual final settlement of 10.10 mm. And the prediction accuracy is improved by 10.70%, which fully verifies the effectiveness of the EMD in the surface settlement prediction model.

## Discussion

### Prediction results under different excitation functions

**Figure 15:**
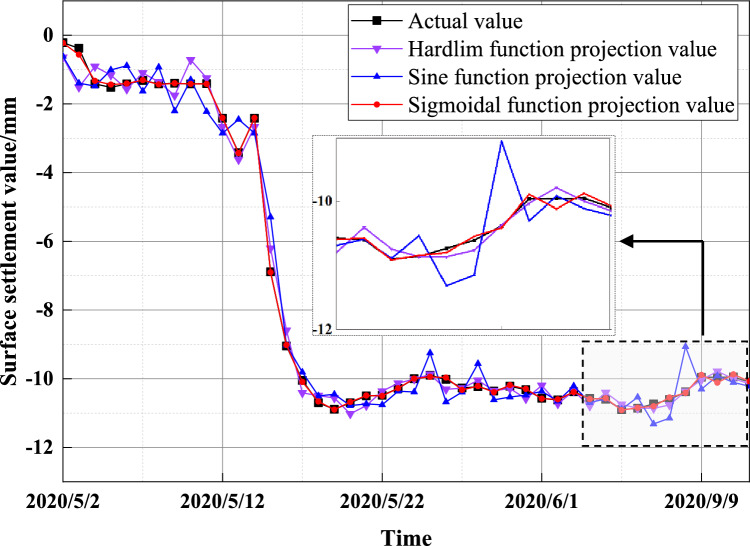
Prediction results with different excitation functions.

Since the output of the upper layer of the ELM prediction model needs to be transformed by the excitation function before it can be input to the next layer, the selection of the excitation function is very important. To compare the effects of different excitation functions on the prediction performance of the model, the commonly used “Hardlim function”, “Sigmoid function” and “Sine function” are selected. The measured values of monitoring point SD1030-5 of the section are used as an example for analysis. Table 3 shows the model errors when different excitation functions are selected, and Fig. 15 shows the prediction results when different excitation functions are selected.

**Table 3:** Comparison of prediction models under different excitation functions.

Excitation functions	MSE	RMSE	MAPE
Sigmoid	0.0026	0.0513	0.0144
Hardlim	0.2739	0.5233	0.1345
Sine	0.1012	0.3181	0.1079

The prediction accuracy of the Sigmoid function is the best when the model parameters are set equal, while the prediction accuracy of the Hardlim function is the worst. When the Sine function is used, the prediction results are in between. In summary, the Sigmoid function is better overall for model prediction in this paper, and the model generalization ability is better.

Table 3 shows that the MSE, RMSE, and MAPE of the prediction model using the Sigmoid function are reduced by 27.13%, 47.20%, and 12.01%, respectively, compared to those using the Hardlim function. The prediction models with the Sine function are reduced by 9.86%, 26.68%, and 9.35%, respectively, compared to the prediction model with the Sine function. Different excitation function choices produce different effects on the surface settlement prediction results, and this paper verifies that the accuracy of the surface settlement prediction model is higher when the Sigmoid function is used as the excitation function.

### Universal adaptability of EMD-CASSA-ELM intelligent predictive model

**Figure 16:**
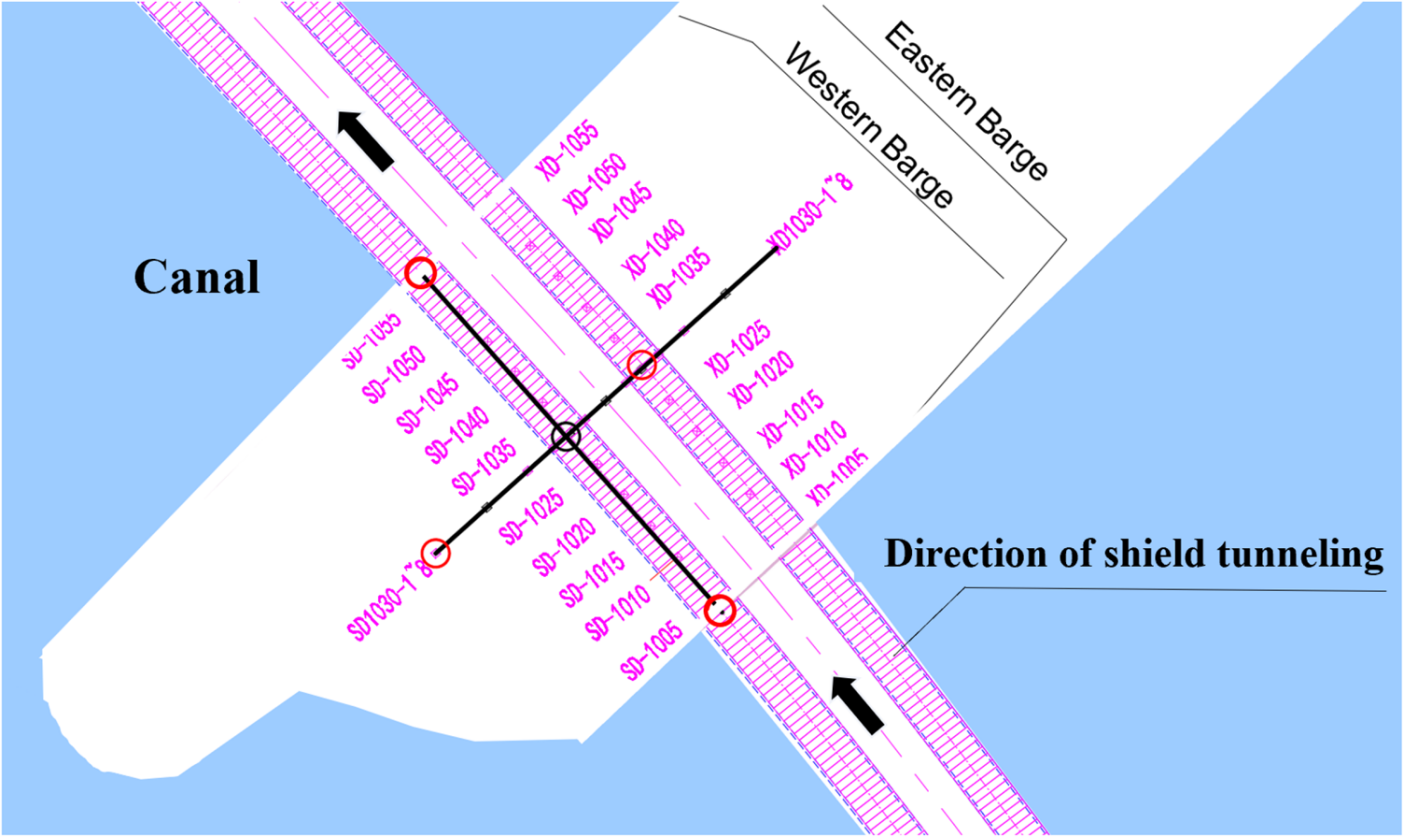
Monitoring point layout.

To verify the general adaptability of the model, surface settlement values at different monitoring sections were predicted and analyzed. Four monitoring points of this shield tunnel under the canal were selected for additional validation, namely monitoring points XD1030-5 on the right line, and three monitoring points SD1005, SD1030-1, and SD1055 on the left line. The four monitoring points were distributed on the orthogonal axis centered on the left shield tunnel monitoring point SD1030-5, which can better reflect the monitoring data at different spatial distribution locations, as shown in Fig. 16.

**Figure 17:**
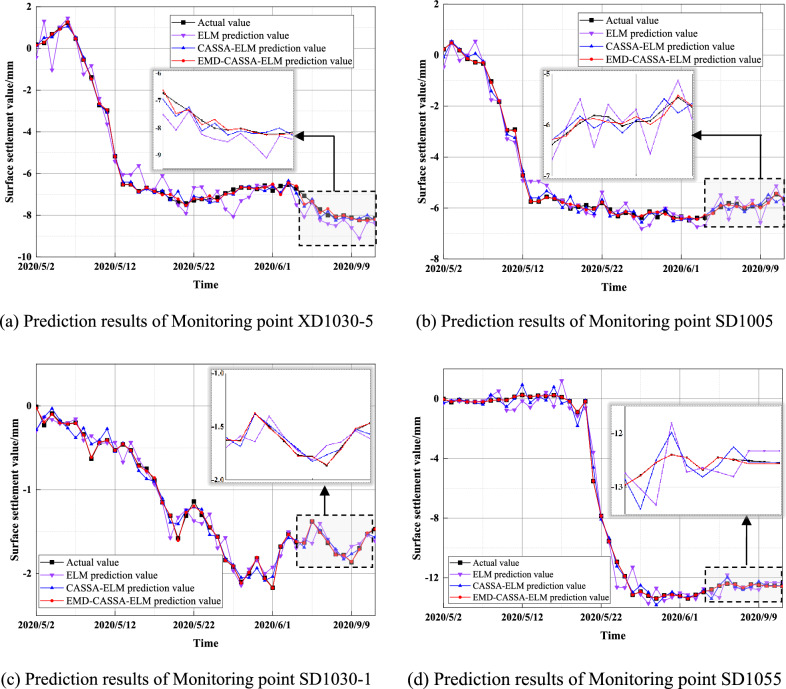
Prediction results of different monitoring points.

The EMD-CASSA-ELM surface settlement predictions were performed for each of the four monitoring sites and compared with the ELM model and CASSA-ELM model, and it was found that the predicted results of the EMD-CASSA-ELM model were closer to the actual values. Figure 17 shows the comparison of the four monitoring sites under the three models.

The three error evaluation indicators of MSE, RMSE, and MAPE were calculated for the four monitoring sites. It was found that the predicted results of the monitoring points at four different locations using the EMD-CASSA-ELM model were all better. Among them, monitoring points SD1055 and SD1005, both of which are located at the monitoring point directly above the left line of the shield tunnel boring, have similar settlement change trends as monitoring point SD1030-5. And the improvement effect predicted by the EMD-CASSA-ELM model is more obvious. For the monitoring point SD1055, the calculation results depict that compared with ELM, the MSE, RMSE, and MAPE using the EMD-CASSA-ELM prediction model were reduced by 41.47%, 62.87%, and 54.78%, respectively. And the CASSA-ELM prediction model was reduced by 30.91%, 31.89%, and 13.07%, respectively, which describes that the EMD-CASSA-ELM model has great improvement and effectiveness. Table 4 fully illustrates the general adaptability of the EMD-CASSA-ELM model in predicting surface settlement in shield tunnels at different spatial locations, which can generally reflect the trend of surface settlement.

**Table 4:** Comparison of evaluation indicators of different monitoring points.

Monitoring point	Model	MSE	RMSE	MAPE
XD1030	ELM	0.3155	0.5617	0.1481
	CASSA-ELM	0.0539	0.2321	0.0548
	EMD-CASSA-ELM	0.0188	0.1372	0.0219
SD1005	ELM	0.1337	0.3656	1.4533
	CASSA-ELM	0.0359	0.1894	0.2300
	EMD-CASSA-ELM	0.0048	0.0694	0.0132
SD1030-1	ELM	0.0182	0.1348	0.1472
	CASSA-ELM	0.0075	0.0866	0.1576
	EMD-CASSA-ELM	0.0003	0.0164	0.0320
SD1055	ELM	0.4149	0.6441	0.5574
	CASSA-ELM	0.1058	0.3252	0.6881
	EMD-CASSA-ELM	0.0002	0.0154	0.0096

## Conclusion

In this paper, a combined EMD-CASSA-ELM prediction model is proposed for predicting the surface settlement of shield tunnels. The model uses empirical mode decomposition to decompose the surface settlement sequence into fluctuation components and trend of components. The CASSA-ELM model is used to reconstruct the prediction of each component to obtain the final settlement. The main conclusions obtained are as follows:

1. The original settlement sequence is decomposed using the empirical mode decomposition method to obtain different IMFs and residual components. Thus, the original sequences with different regular fluctuation sequences or trend sequences existing in the original sequences are decomposed step by step. And the effectiveness of the empirical mode decomposition in the surface subsidence prediction model is verified.
2. A chaotic adaptive sparrow search algorithm is proposed, and the effectiveness of the CASSA-optimized extreme learning machine model for surface subsidence prediction is fully verified. The prediction results after optimizing the limit learning machine using the chaotic adaptive sparrow search algorithm are better than the ELM and SSA-ELM models.
3. The influence of the choice of the excitation function on the prediction performance of the model is explored in depth. It is found that the Sigmoid excitation function is more suitable for the EMD-CASSA-ELM model proposed in this paper, when the model has the best prediction performance and has good generalization ability.
4. This paper proposes a combined prediction model that combines empirical mode decomposition, chaotic adaptive sparrow search algorithm, and extreme learning machine. And the surface settlement prediction is carried out for monitoring points at different spatial locations of the shield tunnel to verify the general adaptability of the model. The application of engineering examples shows that the combined model has high prediction accuracy and generalization ability, which can provide effective guidance for safety monitoring and prediction analysis in shield tunnel construction.

In this paper, EMD-CASSA-ELM model is used to predict the surface settlement of shield tunnel. Among them, meta-heuristic algorithm sparrow search algorithm plays an important role in parameter optimization and improving model prediction accuracy. But in recent years, the fractional order swarming metaheuristics method has been widely applied in many engineering fields. For example, Malik et al.^38^ proposed fractional-order particle swarm optimization (FOPSO) and found that FOPSO converges faster than other fractional-order and standard Psos. Muhammad et al.^39^ proposed a new naturally inspired computing paradigm based on fractional-order synthetic learning particle swarm optimization (FO-CLPSO) for optimal reactive power scheduling problems (ORPD) and compared it with several state-of-the-art methods. Haji et al.^40^ proposed a fuzzy fractional order algorithm based on dynamic particle swarm optimization. This algorithm can be used for optimization task well. Altaf et al.^41^ proposed to introduce the Key Term Separation Technique (KTST) into FO-PSO algorithm to estimate parameters in control autoregressive systems. The performance of KTST-FOPSO is studied in detail according to different fractional order, and the reliability and stability of this model for in-car recognition are verified. In the future, it will be a new development direction to introduce fractional order group heuristic algorithm to shield tunnel safety monitoring and settlement prediction.

## Data Availability

The datasets generated during and/or analyzed during the current study are available from the corresponding author upon reasonable request.
